# Magnetic Resonance-Based Assessment of Optic Nerve Sheath Diameter: A Prospective Observational Cohort Study on Inter- and Intra-Rater Agreement

**DOI:** 10.3390/jcm12072713

**Published:** 2023-04-05

**Authors:** Raffaele Aspide, Giacomo Bertolini, Laura Maria Beatrice Belotti, Luca Albini Riccioli, Francesco Toni, Diego Mazzatenta, Giorgio Palandri, Luigi Vetrugno, Daniele Guerino Biasucci

**Affiliations:** 1 IRCCS Istituto delle Scienze Neurologiche di Bologna, Anesthesia and Neurointensive Care Unit, 40139 Bologna, Italy; 2 Department of Biomedical and Neuromotor Sciences (DIBINEM), University of Bologna, 40126 Bologna, Italy; jackbert@gmail.com (G.B.); diego.mazzatenta@unibo.it (D.M.); 3 IRCCS Istituto delle Scienze Neurologiche di Bologna, Epidemiology and Statistic Unit, 40139 Bologna, Italy; lauramariabeatrice.belotti@ausl.bologna.it; 4 IRCCS Istituto delle Scienze Neurologiche di Bologna, Neuroradiology Unit, 40139 Bologna, Italy; luca.albiniriccioli@isnb.it (L.A.R.); francesco.toni@isnb.it (F.T.); 5 IRCCS Istituto delle Scienze Neurologiche di Bologna, Department of Neurosurgery, 40139 Bologna, Italy; giopalandri@gmail.com; 6 Department of Medical, Oral, and Biotechnological Sciences, University of Chieti-Pescara, 65127 Chieti, Italy; luigi.vetrugno@unich.it; 7 Department of Clinical Science and Translational Medicine, “Tor Vergata” University of Rome, 00133 Rome, Italy; danielebiasucci@gmail.com

**Keywords:** optic nerve sheath diameter, idiopathic normal pressure hydrocephalus, intracranial pressure, magnetic resonance imaging

## Abstract

Background: The measurement of optic nerve sheath diameter (ONSD) as a non-invasive method of estimating intracranial pressure has been widely reported in the literature. However, few studies have evaluated the accuracy of magnetic resonance imaging (MRI) in assessing ONSD measurements, although it is considered a very reliable method, it is not easily repeatable, expensive and is not readily available bedside. Herein, an assessment of the intra- and inter-rater reliability of ONSD assessment using MRI was conducted. Methods: A consecutive, prospective cohort of patients with suspected idiopathic normal-pressure hydrocephalus was analyzed. ONSD MRI measurements of the transverse and sagittal diameters at a distance of 3 mm behind the papilla were evaluated twice each by two expert neuroradiologists. The correlations between MRI examiners were calculated using the concordance correlation coefficient (CCC). Results: Fifty patients were included in the study. ONSD MRI average measurements were substantially higher than clinically expected (>5 mm). Considering intra-rater concordance, only one of the two neuroradiologists achieved an excellent score at CCC. Only a moderate inter-observer CCC for MRI assessment was found at all diameters. Conclusions: The use of a widespread MRI sequence (3D T1) to measure ONSD is not an accurate method since it may overestimate measurements and is dependent upon an operator.

## 1. Introduction

Optic nerve sheath diameter (ONSD) measurement may be a promising method for the non-invasive assessment of intracranial pressure (ICP) [1,2]. The sheath that surrounds the optic nerve is in continuity with the subarachnoid spaces and the cerebrospinal fluid contained therein. As a result, it is susceptible to variation in its diameter in response to ICP fluctuations [3].

According to post-mortem human anatomical dissections, the optic nerve (ON) is characterized by a mean diameter of 3 mm [4], while the optic nerve sheath has an average thickness of 0.4 mm. The subarachnoid space between them measures approximately 0.1 mm [4,5]. Based on those findings, it can be estimated that the ONSD measures approximately 4 mm under physiological conditions [6].

While many comparative studies of non-invasive methods of ICP assessment have been published, there are no large randomized trials, consensus or guidelines available that can identify a gold standard for ONSD assessment [7,8,9].

Among the common non-invasive methods for ONSD assessment, magnetic resonance imaging (MRI), especially with high-resolution sequences, has proven to be an accurate technique [10,11]. However, only a few studies have investigated the reproducibility and accuracy of MRI in ONSD measurements, and those studies have produced slightly conflicting results.

Therefore, in the current study, we aimed at assessing intra-observer and inter-rater reliability of ONSD measurement using MRI in a cohort of patients with idiopathic normal pressure hydrocephalus (iNPH).

## 2. Methods

### 2.1. Study Design, Participants and Setting

This observational, prospective, monocentric study was conducted between February 2018 and April 2019. Patients 18 years of age or older with a suspected diagnosis of iNPH were considered eligible for recruitment. The choice of such a specific patient population was motivated by the availability of a consistent consecutive case series at the enrollment center. Our Institute evaluated patients suspected of having iNPH (PRO-Hydro team) who were referred by neurologists, geriatricians, neurosurgeons and general practitioners. The multidisciplinary team reviewed the patients’ medical records and brain imaging before their assessment. Eligible patients underwent a specific MRI protocol and were then evaluated by neurologists or neurosurgeons in an outpatient visit. Patients with clinical features and neuroimaging indicative of iNPH were admitted to the inpatient iNPH program with Tap Test (TT). The diagnosis was assigned after reviewing all pre-TT clinical data and neuropsychological information, blood/CSF tests and comparing pre- and post-TT during a consensus case conference involving the multidisciplinary team. Based on the diagnosis and taking into consideration comorbidities and vascular risk factors, the multidisciplinary team established eligible patients for shunting. A CT scan was performed 1 day and 1 month after surgery. Both groups of patients, those who underwent Ventricular–Peritoneal shunt and those who did not, were evaluated 6- and 12 months after surgery or after the inpatient iNPH program, respectively. The protocol was thoroughly described elsewhere and all patients met the criteria for probable iNPH following international guidelines [12]. This evaluation was based on clinical and radiological findings and measured against the criteria established by Relkin et al. [13]. In contrast, the exclusion criteria rendered patients ineligible if they had a central nervous system mass, another possible suspected or established primary cause of ICP alteration or a history of ON disease. Additionally, all those who denied or withdrew consent for their enrolment were excluded. Prior to enrolment, during a regularly scheduled neuroradiology session, eligible patients were subjected to a 3-tesla MRI, according to the institutional protocol described below. Technically similar images are well suited to studying intra- and inter-rater reliability in a population without high ICP. The study was conducted in accordance with the Declaration of Helsinki and the general principles of Good Clinical Practice. The Institutional Review Board approved the study to be conducted (Cod. CE 17115). Authors followed the Strengthening the Reporting of Observational Studies in Epidemiology (STROBE) guidelines for cohort studies (http://www.strobe-statement.org (accessed on 20 February 2023)) [14]. 

### 2.2. Demographic and Clinical Variables

The age and sex of all patients were collected as demographic variables. A consecutive cohort of patients was enrolled to reduce the risk of selection bias.

### 2.3. MRI Parameters

A 3-tesla whole-body 32-channel phased-array scanner MRI (Magnetom Skyra; Siemens Healthcare, Erlangen, Germany) was used for image acquisition according to the PRO-Hydro protocol. Sagittal three-dimensional (3D) inversion recovery gradient echo (IR-GRE), magnetization-prepared rapid gradient-echo (MP-RAGE) T1-weighted sequence (repetition time: 2300 ms; echo time: 2.98 ms; flip angle: 9°; thickness: 1 mm; 160 slices; field of view: 256 × 248 mm; matrix: 256 × 248 at 1 mm × 1 mm) was identified by the neuroradiologist (NRD) as the best morphological imaging method for ONSD evaluation due to its contrast between endo-orbital fat and nerve sheath. The measurements were performed on reconstructions carried out both parallel and orthogonal to the ON.

### 2.4. Data Measurement and Endpoints

Two veteran NRDs—LAR and FT—with the same professional experience in MRI reporting discussed the objectives of the study and agreed upon the best strategy for measuring ONSD (projection plane, zoom depth, grayscale, etc.). These physicians performed two serial ONSD measurements on MRI results at two different time points (called t1 and t2) on both eyes. ONSD was measured 3 mm behind the optic disc, in accordance with the findings of the Helmke and Hansen study [1]. All measurements were performed bilaterally. The right and left transverse diameters (TDR and TDL, respectively) and right and left sagittal diameters (SDR and SDL, respectively) were measured. The second blinded assessment (t2) was performed two weeks after the first one (t1) on the same recorded images, and the reliability of their measurements was then calculated. This method of data collection allows the calculation of the composite primary endpoint: the intra-rater agreement (the degree of similarity between t1 and t2 values obtained by the same NRD) and inter-rater agreement (the degree of similarity between the same time point values obtained by the two NRDs) were calculated. To minimize the risk of confirmation bias, a two-week timespan was mandatory between the first and second assessments. Furthermore, each NRD was blinded to the other physician’s measurements.

### 2.5. Statistical Analysis

The patients’ demographic and clinical characteristics were analyzed using descriptive statistics. The results were presented as mean ± standard deviation (SD) or as numerical values (%) unless otherwise specified. The concordance of MRI measurements performed by the same NRD at different times (intra-rater agreement) or by the two NRDs at the same time (inter-rater agreement) was assessed using Lin’s concordance correlation coefficient (CCC) with a 95% confidence interval (CI). Lin’s CCC ranges from −1 to 1, with a value of 0 indicating no agreement and a value of 1 or −1 corresponding to perfect agreement between the two raters or assessments. For values < 0.20, concordance is considered “poor”; for values between 0.20 and 0.80, it is considered “moderate” and for values > 0.80, it is “excellent” [15]. Statistical analysis was performed using Stata statistical software version 14 (StataCorp LLC, College Station, TX, USA).

Because there are very few articles in the literature examining this phenomenon (comparing how NRDs use MRI to evaluate ONSD), it was not possible to generate a power analysis able to define a sample size. Furthermore, due to the learning curve for measuring ONSD via MRI, only two NRDs performed the measurements. Therefore, the present study may not be able to validate the method but can be considered a pilot study.

## 3. Results

Fifty consecutive patients—29 (58%) males and 21 (42%) females—met the eligibility criteria and had a mean age (±SD) of 76 ± 8 years. Details of the mean (±SD) ONSD MRI measurements are reported in Table 1.

NRD intra-rater concordance: The agreement between measurements by the same NRD at two different times was compared for each of the two different diameters for each eye. Intra-rater reliability of NRD 1 and NRD 2 between t1 and t2 using Lin’s CCC are shown in Table 2 and Table 3, respectively. Of the two NRDs involved in the study, only NRD 2 had an excellent CCC; the coefficients of SDR, TDL and SDL were 0.769, 0.808 and 0.791, respectively. The result regarding TDR was graded as moderate (CCC = 0.678). The limits set for CCC are discretionary and should not be interpreted rigidly (for example, “excellent” means >0.75–0.8).

NRD inter-rater concordance: To determine the level of agreement on MRI assessment between the two NRDs, an inter-rater reliability value was determined using Lin’s CCC. The values measured by the two NRDs in the first and second assessments (Table 4 and Table 5, respectively) and the overall mean between these two assessments (Table 6) were used to generate the CCC. Only a moderate inter-rater agreement was found in all the diameters assessed.

## 4. Discussion

In this study, the authors measured ONSD with MRI in a consecutive group of patients with suspected iNPH diagnosis. The mean age of enrolled patients, pathology (iNPH) and type of MRI sequences are homogeneous, which guarantees the reliability of the described results. MRI measurements were substantially higher than clinically expected (>5 mm). This finding can be partially explained by the high average age of the population enrolled in the study. Considering the composite primary endpoint, only one of the two experienced NRDs had excellent intra-rater reliability. In other words, there was a 50% chance—like flipping a coin. Clearly, the results obtained for the calculation of inter-rater reliability are moderate, as none of the diameters reached a CCC of >0.8 (Table 4, Table 5 and Table 6). Our study proves that assessing ONSD using MRI is an operator-dependent technique.

Despite good intra- and inter-rater ONSD MRI agreements reported in previous studies [7,16,17], our data did not show similar strength of accordance (Table 2, Table 3, Table 4, Table 5 and Table 6). In 2008, Thomas Geeraerts and his collaborators studied 38 patients with severe traumatic brain injury (TBI) using ICP monitoring, as well as 36 healthy volunteers. ONSD was measured on a T2-weighted turbo spin-echo fat-suppressed sequence obtained with a 3-tesla MRI. The ONSD in TBI patients with raised ICP (>20 mmHg) was 6.31 ± 0.50 mm; in those with low ICP (<20 mmHg), it was 5.29 ± 0.48 mm; and in healthy volunteers, it was 5.08 ± 0.52 mm. Geeraerts et al. found a significant relationship between ONSD and ICP (r = 0.71, *p* < 0.0001). Enlarged ONSD was a robust predictor of raised ICP (area under the receiver operating characteristic curve = 0.94), with a diameter of 5.82 mm, as the best cutoff, which corresponded to a negative predictive value of 92%. When ONSD was less than 5.30 mm, the predictive value was 100%. In this study, however, the authors only measured the transverse diameter. The slice thickness and interslice spacing were relatively large (4 and 5 mm) and agreement between observers in their measurements of ONSD was relatively poor [7].

Kim et al. focused on 314 healthy Korean adults and identified a strong correlation between ONSD measurement in MRI (mean value = 4.71 mm) and eyeball transverse diameter (EDT) (mean value = 22.24 mm). The study also calculated a ratio between these two diameters, such that ONSD/ETD had a mean value of 0.22 [18].

Zheng et al. studied 145 healthy volunteers and found a good correlation between ONSD in MRI and body mass index (BMI). The cursors of the caliper were placed on the outer contour of the optic nerve sheath, generating another index: ONSD delta = (ONSD − 0.045  × BMI) [19]. In contrast, in Dogan et al.’s study of children, ONSD assessment was performed between the inner edges of the dura surrounding the optic nerve. In addition, in the pediatric world, Janthanimi et al. aimed to identify normal values of ONSD in children under 4 years of age. They measured ONSD in a axial, T2-weighted sequence between the inner borders of the surrounding dura and found a mean value of 5 mm (95% CI 4.9–5.1) [20].

In MRI images, even after the administration of a contrast medium, the visualization quality of the perineural vessels remained low [21]. As discussed in the background section, the diameter of ONSD assessed post-mortem is about 4 mm [6]. It is possible that MRI images tend to overestimate the diameter, since the boundary of the gray sheath lathes is not clearly distinguishable from the area of perineural tissue surrounding the small vessels, even in coronal sequence.

The discrepancies between our results and the data in the literature could be related to differences in the adopted MRI sequence compared to previous studies [18,19]. Over the course of the diagnostic process, some of the enrolled patients underwent a high-volume tap test. CSF opening pressure was also measured. None of the patients developed high ICP. The measurements performed in the present study were based on standard protocols contextualized in clinical practice. Previously, other authors used dedicated experimental sequences—e.g., constructive interference in steady state (CISS), fast imaging employing steady-state acquisition (FIESTA), driven equilibrium radio frequency reset pulse (DRIVE)—or specific planes defined perpendicular or parallel to the orbital axis [8]. These kind of sequences are substantially more difficult to achieve in a high-ICP evaluation context. In the design of this study, the 3D MP-RAGE T1-weighted images were selected to provide higher contrast resolution between optic nerve sheath and orbital fat. Despite the relatively high resolution, however, a significant loss in image definition was observed upon zooming in to the region of interest. The edges of the optic nerve sheath appeared blurred and complicated the analysis of adjacent adipose tissue.

Recent studies, some of them based on experimental models, question the validity of ONSD as a non-invasive measure of ICP [22]. After the application of very high pressures (45–65 mmHg), the sheath does not appear to return to its basal diameter, as if some viscoelastic mechanism is altered [23]. Because of these deformation mechanisms, a threshold for the presence of elevated ICP cannot be identified [24]. The value of ONSD in subsequent measurements and at non-extreme intracranial pressures, especially in the acute phase, remains valid.

The great diffusion in recent years of the ultrasound (US) method for the measurement of ONSD, repeatable, non-invasive, inexpensive and available bedside, still denotes many limitations due to the operator-dependent method and there is little agreement on the cut-off values compared to invasive measurement of ICP. In the same authors’ work, which compares measurements of ONSD performed with US and MRI, the data contradict the idea that measurement with US is absolutely imprecise and too operator-dependent [25]. This team of authors is working on implementing a “bundle” capable of reducing the measurement error of ONSD with US [26].

## 5. Limitations

There are some important limitations in the present study. First, for reasons specified in the Statistical Analysis section, a power analysis was not carried out for the calculation of a correct sample size. Second, since the measurement was not routine for NRDs, a learning curve was required and it was therefore only possible to compare two NRDs. Third, this is a monocentric study. Finally, these results cannot be generalized because the iNPH population studied in the present work could be highly selective.

## 6. Conclusions

Optic nerve sheath thickness is a promising non-invasive measure of intracranial pressure, but unfortunately there is no method that is a gold standard. Even magnetic resonance imaging is an unreliable method from the measurements performed by expert neuroradiologists with non-dedicated standard sequences. Poor-to-moderate intra- and inter-rater reliability was demonstrated in a patient population with suspected iNPH. These findings will require confirmation by future better-designed studies, with adequate sample sizes of both patients and assessors.

## Figures and Tables

**Table 1:** Magnetic Resonance Imaging (MRI) measurement values for each assessment. The reported measurements are expressed in mm Standard Deviation (SD). NRD: neuroradiologist; SDL: sagittal diameter left; SDR: sagittal diameter right; t1: first assessment; t2: second assessment; TDL: transverse diameter left; TDR: transverse diameter right.

	TDR	SDR	TDL	SDL
NRD 1—t1	5.66 ± 0.69	6.00 ± 0.89	5.57 ± 0.83	5.77 ± 0.94
NRD 1—t2	5.87 ± 0.71	6.37 ± 0.88	5.93 ± 0.87	6.12 ± 0.87
NRD 2—t1	6.34 ± 0.70	6.45 ± 0.69	6.28 ± 0.77	6.32 ± 0.76
NRD 2—t2	6.50 ± 0.68	6.56 ± 0.66	6.47 ± 0.70	6.47 ± 0.76

**Table 2:** Intra-rater agreement in ONSD Magnetic Resonance Imaging (MRI) assessment for NRD 1.

NRD 1— Assessment t1 vs. t2	TDR	SDR	TDL	SDL
CCC (95% CI)	0.682 (0.537, 0.827)	0.406 (0.187, 0.624)	0.600 (0.433, 0.768)	0.550 (0.367, 0.734)

A CCC of <0.20 was considered “poor” and >0.80 was considered “excellent. CI Confidence Interval NRD: neuroradiologist; CCC: Lin’s concordance correlation coefficient; TDR: transverse diameter right; SDR: sagittal diameter right; TDL: transverse diameter left; SDL: sagittal diameter left.

**Table 3:** Intra-rater agreement in ONSD MRI assessment for NRD 2.

NRD 2— Assessment t1 vs. t2	TDR	SDR	TDL	SDL
CCC (95% CI)	0.678 (0.530, 0.827)	0.796 (0.693, 0.898)	0.808 (0.715, 0.902)	0.791 (0.688, 0.895)

A CCC of <0.20 was considered “poor” and >0.80 was considered “excellent.” CI Confidence Interval NRD: neuroradiologist; CCC: Lin’s concordance correlation coefficient; TDR: transverse diameter right; SDR: sagittal diameter right; TDL: transverse diameter left; SDL: sagittal diameter left.

**Table 4:** Inter-rater agreement in ONSD MRI assessment between the two neuroradiologists in the first assessment (assessment t1, NRD 1 vs. NRD 2). Inter-rater agreement comparing the first MRI assessment of the two neuroradiologists.

Assessment t1—NRD 1 vs. NRD 2	TDR	SDR	TDL	SDL
CCC (95% CI)	0.441 (0.283, 0.598)	0.574 (0.415, 0.732)	0.515 (0.364, 0.665)	0.645 (0.513, 0.776)

A CCC with 95% CI of <0.20 was considered “poor” and >0.80 was considered “excellent”. Only a moderate inter-rater agreement was found. NRD: neuroradiologist; CCC: Lin’s concordance correlation coefficient; CI Confidence Interval TDR: transverse diameter right; SDR: sagittal diameter right; TDL: transverse diameter left; SDL: sagittal diameter left.

**Table 5:** Inter-rater agreement in ONSD MRI assessment between the two neuroradiologists in the second assessment (assessment t2, NRD 1 vs. NRD 2). Inter-rater agreement comparing the second MRI assessment of the two neuroradiologists.

Assessment t2—NRD 1 vs. NRD 2	TDR	SDR	TDL	SDL
CCC (95% CI)	0.387 (0.212, 0.561)	0.436 (0.223, 0.648)	0.556 (0.401, 0.711)	0.567 (0.391, 0.742)

A CCC with 95% CI of <0.20 was considered “poor” and >0.80 was considered “excellent”. Only a moderate inter-rater agreement was found. NRD: neuroradiologist; CI Confidence Interval CCC: Lin’s concordance correlation coefficient; TDR: transverse diameter right; SDR: sagittal diameter right; TDL: transverse diameter left; SDL: sagittal diameter left.

**Table 6:** Inter-rater agreement in ONSD MRI assessment between the mean of the two assessments for the two NRDs (mean of assessments t1 and t2, NRD 1 vs. NRD 2). Inter-rater agreement in MRI comparing the mean values of assessments I and II between the two neuroradiologists.

Mean of Assessment t1 and t2— NRD 1 vs. NRD 2	TDR	SDR	TDL	SDL
CCC (95% CI)	0.452 (0.301, 0.603)	0.645 (0.497, 0.793)	0.588 (0.453, 0.723)	0.697 (0.576, 0.819)

A CCC with 95% CI of <0.20 was considered “poor” and >0.80 was considered “excellent”. Only a moderate inter-rater agreement was found. NRD: neuroradiologist; CCC: Lin’s concordance correlation coefficient; TDR: transverse diameter right; SDR: sagittal diameter right; TDL: transverse diameter left; SDL: sagittal diameter left.

## Data Availability

The data that support the findings of this study are available from the corresponding author, [RA], upon reasonable request.

## Abbreviations

ONSD	Optic Nerve Sheath Diameter
ICP	Intracranial Pressure
ON	Optic Nerve
MRI	Magnetic Resonance Imaging
iNPH	idiopathic Normal Pressure Hydrocephalus
TT	Tap Test
STROBE	Strengthening the Reporting of Observational Studies in Epidemiology
3D	Three-dimensional
IR-GRE	Inversion recovery gradient echo
NRD	Neuroradiologist
SDL	Sagittal Diameter Left
SDR	Sagittal Diameter Right
TDL	Transverse Diameter Left
TDR	Transverse Diameter Right
SD	Standard Deviation
CCC	Concordance Correlation Coefficient
CI	Confidence interval
TBI	Traumatic Brain Injury
EDT	Eyeball Transverse Diameter
BMI	Body Mass Index
CISS	Constructive interference in steady state
FIESTA	Fast imaging employing steady-state acquisition
DRIVE	Driven equilibrium radio frequency reset pulse
US	Ultrasound
